# Bioresorbable Magnesium-Based Alloys as Novel Biomaterials in Oral Bone Regeneration: General Review and Clinical Perspectives

**DOI:** 10.3390/jcm10091842

**Published:** 2021-04-23

**Authors:** Valentin Herber, Begüm Okutan, Georgios Antonoglou, Nicole G. Sommer, Michael Payer

**Affiliations:** 1 Department of Dentistry and Oral Health, Division of Oral Surgery and Orthodontics, Medical University of Graz, Billrothgasse 4, 8010 Graz, Austria; antonoglou.georgios@gmail.com (G.A.); mi.payer@medunigraz.at (M.P.); 2 Department of Orthopaedics and Traumatology, Medical University of Graz, Auenbruggerplatz 5/6, 8036 Graz, Austria; beguem.okutan@medunigraz.at (B.O.); nicole.sommer@medunigraz.at (N.G.S.)

**Keywords:** magnesium, alloy, biomaterial, bone regeneration

## Abstract

Bone preservation and primary regeneration is a daily challenge in the field of dental medicine. In recent years, bioresorbable metals based on magnesium (Mg) have been widely investigated due to their bone-like modulus of elasticity, their high biocompatibility, antimicrobial, and osteoconductive properties. Synthetic Mg-based biomaterials are promising candidates for bone regeneration in comparison with other currently available pure synthetic materials. Different alloys based on Mg were developed to fit clinical requirements. In parallel, advances in additive manufacturing offer the possibility to fabricate experimentally bioresorbable metallic porous scaffolds. This review describes the promising clinical results of resorbable Mg-based biomaterials for bone repair in osteosynthetic application and discusses the perspectives of use in oral bone regeneration.

## 1. Introduction

### 1.1. Introduction to Magnesium

Magnesium (Mg) is the lightest structural metal and increased interest within automobile and aerospace industries since the late 1990s with the requirement of weight-saving metals [1]. In electric and informatic industries, Mg-based alloys have been widely used due to their advantages, e.g., the high specific strength, environmental friendliness, and good thermal and electrical conductivity [2].

Mg is the fourth most abundant cation element found within the human body, and involved in more than 300 known cell enzymatic reactions [3]. Mg plays an important role in mitochondrial activity, protein, and nucleic synthesis, and therefore in energy metabolism and cell proliferation [4,5]. With approximately 21 g to 28 g of Mg in a healthy adult, over 50% is stored in the bone [4]. Mg balance depends on the storage in bone, the intestinal uptake, and the renal excretion [6]. The kidney is the principal organ responsible in Mg homeostasis [7]. A disrupted Mg homeostasis in associated with severe pathological conditions, such as hypo- or hypermagnesemia [8].

Mg and its alloys have gained interest in the field of medicine due to their high bioresorbability, as well as their bone-liked elastic modulus to bone. A clear advantage of bioresorbable materials in daily clinics is the avoidance of second stage surgeries and reduction of associated comorbidities. Mg corrodes in the physiological environment and releases species such as Mg^2+^ ions (Mg^2+^), alloying elements, H_2_ gas, and OH^−^ [9]. In an alkaline environment, magnesium hydroxide Mg(OH)_2_ is deposited on the Mg matrix and forms a protective layer [10]. If the corrosive environment contains more than 30 mmol·L^−1^ of chlorides (Cl^−^), Mg(OH)_2_ is converted to magnesium chloride (MgCl_2_), which is highly soluble [11]. The intermediate corrosion products can be absorbed in the physiological environment or digested by macrophages [12]. During the corrosion of Mg, H_2_ is released in the surrounding tissues. Song considered that a H_2_ evolution rate of 0.01 mL/cm^2^/day can be tolerated by the human body [13]. Liu et al. recently showed that H_2_ have a positive effect on bone regeneration while inhibiting the osteoclastogenesis of mouse bone marrow mononuclear cells [14]. Mg^2+^ are physiologically present in the human body and participate in metabolic reactions and biological mechanisms. No critical toxic limits have been reported concerning released Mg^2+^ as their excess is easily excreted via urine [15].

The corrosion of Mg and its alloys depends on several factors. The heterogeneity, metal purity and microstructure of the alloy will influence the corrosion [16]. Additionally, mechanical loads can form stress corrosion cracking and/or corrosion fatigue that can lead to implant failure [17]. After implantation, the pH of the surrounding tissues and the vascularization of the peri-implant zones are factors influencing Mg corrosion [18]. Some corrosion conditions such as pH values of 7.4–7.6 and high Cl^−^ concentration leads to a fast corrosion of the Mg-based materials. Uncontrolled Mg corrosion possibility generates (i) an increase of the local pH by releasing OH^−^ ions, (ii) changes of the mechanical behaviour, as well as (iii) the release of H_2_ in the surrounding tissue [19,20]. In this way, the controlled corrosion of Mg is the key for further clinical application.

This article focusses on the future application of bioresorbable Mg-based materials in oral bone regeneration. To understand the interest of Mg in this use, we first describe the need of bioresorbable materials and review Mg and Mg-based alloys already investigated in clinical trials. To have an overview on the effectiveness and safety of Mg, we discuss previous clinical trials evaluating Mg and its alloy upon bone repair in the field of orthopaedic and orthognathic surgeries. The limitations of pure synthetic material for bone regeneration and the advantages of Mg are also discussed. Finally, perspectives of Mg’s application in oral bone regeneration are exposed.

### 1.2. First Clinical Implantation of Mg for Orthopaedic Surgery

Since the 19th century, Mg attracted attention from biomedical researchers and clinicians. The first clinical evaluation of Mg was performed in 1900 in Europe. Payr et al. proposed and investigated Mg as bioresorbable material for musculoskeletal surgical application [21,22]. Then, in 1906 in France, Albin Lambotte and Jean Verbrugge investigated the clinical use of pure Mg for osteosynthesis by implanting pure Mg discs associated with steel screws into the fibula. They reported post-operative massive subcutaneous gas formations and local swelling resulting from the formation of gas accelerated by the steel screws, which have a higher corrosion potential than Mg [23,24]. After this failure, Lambotte and Verbrugge inserted pure Mg nails to fix supracondylar fractures of children with uncomplicated functional restoration of the joint [25]. The first alloying system based on Mg, zinc, and aluminium, was pre-clinically and clinically investigated for children transdiaphyseal humerus fracture approximately two decades after the first use of Mg by the French researchers [23]. Around 1938, McBride who firstly criticized the use of Mg plates for bone reconstruction, suggested and highlighted new surgical approaches with Mg screws, including bone drilling before screw insertion [26]. A few years later, Maier reported two clinical cases with humerus fractures treated with spindle shaped pins which were associated with promising clinical outcomes [27]. In 1948, Troitskii (Трoицкий) and Tsitrin (Цитрин) communicated the successful use of screws and plates based on Mg and a small amount of cadmium in cases of pseudarthrosis [28]. Znamenskii also alloyed Mg with a small amount of aluminium, and reported good clinical outcomes [29].

From the middle of the 19th century to the beginning of the 20th, no clinical trials were reported in the literature. This lack of interest could be explained by the introduction and successful use of non-absorbable materials such as titanium alloys (Ti), which exhibit excellent osteoconductive, mechanical, and biocompatible properties. Nonetheless, in the last decade, and in parallel to the development of new and active biomaterials, interest has been renewed in Mg.

### 1.3. Clinical Need of Bioresorbable SYNTHETIC Materials in the Field of Oral Bone Regeneration

Different physiological and pathological situations such as tooth extraction, injuries, diseases, use of certain drugs, including bisphosphonates, or surgeries can lead to vertical and/or horizontal bony defects. Despite the natural healing process, bone augmentation is essential in many situations to allow implant placement and subsequent dental reconstruction. Just after blood transfusion, bone grafting procedures are the second most frequent tissue transplantation [30]. Bone graft represents more than two million surgical procedures annually in the world with a market size valued at USD 2.78 billion in 2020 [31,32].

Autogenous bone is still considered gold standard for bone repair, since the graft is harvested from the same patient and no complications of the immune system may be expected [33]. However, important bone resorption has been reported after the clinical use of autogenous grafts [34,35]. Intraoral block grafts are commonly taken from the mandibular symphysis or ramus and are associated with less donor site morbidities compared with those extraorally harvested. However, a limited volume of intraoral grafts has to be considered when reconstructing large intraoral defects [36,37,38]. To avoid a second surgical site and reduce possible patient discomfort, three other classes of grafts were differentiated and developed: allografts, xenografts, and the synthetic grafts (alloplasts). Allografts are harvested from genetically non-identical members of the same species [39]. The risk of disease transmission exists, but is negligible and, as an example, is estimated at 1 in 1.6 million for the human immunodeficiency virus (HIV) [40]. Xenografts, derived from non-human species, mainly bovine, are also osteoconductive and are widely used in daily practice [39]. Bovine-derived bone substitutes (particulate and blocks) have successfully been documented in clinical application for the treatment of bone defects and ridge augmentation procedures [41,42,43]. However, due to the origin of xenografts, patients’ religious beliefs as well as dietary restrictions have catalysed the search for alternative materials [44]. In this context, synthetic resorbable bone substitutes that are partially or completely replaced after the healing process by newly formed bone and are devoid of biological material (either collagen or protein) have gained interest in research.

In cases of guided bone regeneration (GBR) and guided tissue regeneration (GTR) which are widely used to treat alveolar bone defects, bioresorbable membranes aim to interfere the ingrowth of soft tissue or epithelium into the defect site and promote periodontal tissue or bone regeneration without removal of the membrane [45,46]. However, even nowadays in the reconstruction of severe critical size defects the application of non-resorbable membranes and/or synthesis, screws or plates may still be seen in daily clinics. Thus, there still seems to be an urgent clinical need for research and reliable data on bioresorbable materials used in GBR/GTR procedures, as they do not require any second stage surgery and may reduce related-costs [47].

In addition, after orthognathic surgery, metallic fixation biomaterials are commonly made of Ti. Although these devices offer superior mechanical properties, they are bioinert, and permanently remain as a foreign material in the body. Surgical site infections (SSI) occur in 1.4% to 33.4% of all cases [48].

## 2. Mg and Its Alloys

The degradation rate of Mg and its alloys is the main limitation in the clinical field. This degradation rate of implants should match with bone healing rate. To improve corrosion resistance of Mg-based implants, purification and material alloying are useful methods. Several Mg-based alloys (i.e., Mg-Ca alloy, Mg-Zn alloy, Mg-Si alloy, Mg-REE alloy) have been investigated in vitro and in vivo to be used for clinical applications.

The following section will review widely used Mg-based biomaterials in clinics, including their composition, mechanical properties, and pre-clinical studies. The detailed pre-clinical studies of these alloys were summarized in Table 1.

### 2.1. Pure Mg

Pure Mg is the simplest form of material among all other bioresorbable materials. Since Mg has a fast corrosion rate when compared to other metals, alloying pure Mg with other elements can increase the galvanic corrosion of Mg-based alloy [50,65]. Additionally, degradation rate of pure Mg is influenced by impurities (i.e., Fe, Ni, Cu) [50]. Therefore, the corrosion rate of high-purity (HP) Mg (99.99%), which has very low amounts of impurities, is lower than many other Mg-based alloys [50]. Low mechanical strength limits the usage of these biomaterials as heavy load-bearing implants. Although it can limit the usage of pure Mg implants, many pre-clinical studies confirmed that pure Mg can promote new bone tissue formation, which makes them a great candidate for low load bearing implants [49,50,66].

Han et al. conducted in vitro and in vivo studies on high-purity Mg (HP Mg) that shows a great potential as internal fixation devices for femoral intracondylar fractures. In vitro analysis indicated that HP Mg had uniform corrosion behaviour and promoted the expression level of osteogenic related genes (i.e., osteopontin, alkaline phosphatase, RUNX2). Similarly, good osseointegration and fracture healing were observed in surrounding HP Mg screws in rabbit femurs 8 weeks after implantation [50]. In another pre-clinical study, HP Mg screws were applied in the rabbit model of anterior cruciate ligament (ACL) reconstruction and compared to Ti screws. Biomechanical properties of HP Mg screws were comparable with Ti screws. Histological analysis revealed formation of distinct fibrocartilage transition zones at the tendon-bone interface within the HP Mg group, whereas a disorganized fibrocartilage layer was observed in Ti group 12 weeks after implantation. Moreover, gene expression analysis showed increased BMP-2 and VEGF levels in the HP Mg group compared to Ti group. These results indicated that Mg^2+^ ions released from HP Mg promoted fibrocartilaginous enthuses regeneration [67]. Besides the effect on bone healing, Zhang et al. investigated in vivo degradation and histocompatibility of pure Mg and Mg-6Zn wt.% alloy into the bladders of Wistar rats. Both implant materials exhibited good histocompatibility and no adverse effect was found in bladder, liver, and kidney tissues 2 weeks after implantation. In terms of degradation, pure Mg degraded slower than the Mg-based alloy in both in vitro and in vivo [68].

### 2.2. Mg-REE Alloy

In order to decrease the degradation rate of pure Mg, rare earth elements (REE) are predominantly added to improve the corrosion resistance and mechanical strength of Mg-based biomaterials. Yttrium (Y), scandium (Sc), gadolinium (Gd), zirconium (Zr), and neodymium (Nd) are intensively studied. WE43 alloy (Mg-3.5% Y-2.3% Nd-0.5% Zr, wt.% and trace amount of other REEs) has been used in many medical applications as pins, screws, and cardiovascular stents [69].

Oshibe et al. investigated degradation and biocompatibility of the anodized and monolithic cylindrical WE43 implants in Sprague Dawley rats. Therefore, WE43 was implanted into the tibia and evaluated over a one-year long-term follow-up period. One year after surgery, bone maturation progressed, and lamellar bone structure developed around the implant in both groups. The WE43 implants showed good long-term stability and biocompatibility in bone tissue [57]. Besides, Levorova et al. implanted WE43 and Ti screws into the tibia of rabbits. A significant volume loss of WE43 screws was observed between the 12th and 16th weeks after implantation. Histological analysis demonstrated comparable bone healing around the WE43 and Ti screws without any adverse effect [56]. Moreover, biocompatibility and mechanical properties of WE43 are still being improved and tested in many preclinical studies. For instance, Torrini et al. compared biological response and degradation pattern of WE43 (as-cast) and artificially aged Mg alloy (WE43-T5) in an intraosseous mandible sheep model. As control, Ti screws were used. At 24 weeks, histomorphological analysis showed that WE43-T5 alloy had higher degradation rate and enhanced bone remodelling compared to WE43 (as-cast). Therefore, the developed WE43-T5 alloy is a suitable candidate as an endosteal bone screw [58]. Another in vivo study carried out by Byun et al. concluded that extruded WE43 was suitable to be used for mid-facial application. Extrusion technique improved the mechanical strength of WE43 and promoted the new bone tissue formation around the plates and screws [55].

MgYREZr alloy is the first clinically approved Mg-based biomaterial similar to the WE43 alloy. Many in vivo studies confirmed that this alloy type has good biocompatibility [59,70]. Waizy et al. implanted MgYREZr screws into the bone marrow cavity of rabbit femoral bones (New Zealand White rabbit) to investigate the local effects on bone tissue and systemic reactions 1-, 12-, and 52-weeks after implantation. Histological analysis revealed moderate bone formation around screws without a fibrous capsule. There were no adverse effects on lung, liver, intestine, kidneys, pancreas, and spleen tissue samples according to histopathological evaluations. This study revealed that MgYREZr alloy have good biocompatibility and osteoconductivity [59].

Mg-Nd-Zn-Zr alloy (Jiaoda BioMg, denoted as JDBM) is another Mg-REE based alloy produced by Zhang et al. [71]. Developed new type Mg-REE based alloy has better mechanical properties as well as sufficient corrosion resistance. Therefore, this alloy type is investigated in many pre-clinical studies as well [72,73]. Guan et al. coated Mg-Nd-Zn-Zr (JDBM) with brushite and tested for mandibular bone repair both in vitro and in vivo. Brushite-coated JDBM screws were implanted into mandible bones of rabbits for 1, 4, and 7 months, respectively, using 316 L stainless steel screws as a control group. This coated alloy not only showed a slow degradation rate at an early stage but also induced osteogenesis of the mandibular bone, indicating a great potential for mandibular repair [60,61]. Another designed Mg-Nd-Zn-Zr alloy (NZK) was investigated to analyse degradation behaviour and biocompatibility at early time points. Alloys were implanted into the New Zealand white rabbit’s femur and compared with Ti alloy and sham-operated group as controls. A good biocompatibility was observed, and the degradation rate was calculated as 0.66 and 0.48 mm/year at 28 and 56 days, respectively. Wang et al. suggested that designed NZK alloy can degrade gradually in rabbit femur [61].

Despite the promising preclinical results, some questions remain concerning the safety of REE. In fact, these elements do not naturally exist in the body, and their long-term effects are still unknown. Some negative in vitro effects were shown on effects on murine fibroblast and osteoblast cells [74], and on human cell lines [75]. To date, no biocompatible complications were shown in vivo, maybe due to the relatively small amount of REE present in these alloys.

### 2.3. Mg-Ca-Zn Alloy

Compared to previous alloys, alloys made with Mg, these alloys are mainly focusing on nutrient elements, such as calcium (Ca) and zinc (Zn). Ca is one of the main elements in the human body, especially for bone and teeth, and plays an important role in many cellular signalling pathways. Moreover, many studies revealed that Ca can promote bone healing; therefore, alloying Mg and Ca might be a positive effect on bone healing [76]. On the other hand, Zn, which is an essential trace element in the human body, takes part in many biological processes such as wound healing, catabolism of carbohydrates, immunological response, bone development, and growth [77]. Moreover, Zn can improve the mechanical strength of Mg alloys depending on Zn concentration. Cai et al. revealed that up to 5% of Zn can increase the strength of Mg based alloys. Therefore, approximately 4% of Zn showed best mechanical properties among other concentrations [78].

Wong et al. studied the biocompatibility and osteogenic capacity of Mg_60_Zn_35_Ca_5_ bulk metallic glass in a rabbit tendon–bone interference fixation model for a period of up to 24 weeks. Conventional Ti alloy (Ti_6_Al_4_V) and polylactic acid (PLA) were used as controls. Results showed that this biomaterial had sufficient biocompatibility and promoted new bone tissue formation after 24 weeks when compared to Ti and PLA groups [79]. ZX00 (MgZnCa; <0.5 wt.% Zn and <0.5 wt.% Ca) is recently developed Mg-Zn-Ca alloy system, especially for children [80]. Grün et al. investigated ZX00 degradation and bone formation in rat and sheep models. In both animal models, ZX00 exhibited homogenous degradation behaviour. New bone formation was observed around implants after 24 weeks in rat femurs, and osseointegration was observed in both models [63]. In another study, ZX00 screws were implanted into a fractured bone of a growing sheep model and compared with the non-fracture control group in terms of degradation behaviour and fracture healing. Results revealed that fractures healed after a maximum of 12 weeks, and degradation behaviour was comparable with control group [64]. Therefore, both studies confirmed that ZX00 is a great candidate for bone-implant applications.

As summarized in Table 1, in vivo degradation rate can be calculated using volume loss during the degradation of Mg. This rate varies depending on the type of alloys and the type of animal model. For rats, the small animal model is widely used, it varies from 0.41 to 0.40 mm/year for pure Mg [52,54] and 0.08 mm/year for ZX00 [63].

### 2.4. Material Specificities for Oral Application

According to these preliminary results, Mg and its alloys exhibit excellent in vivo biocompatibility, and gradual bioresorbability while promoting bone formation after implantation. Biomaterials developed for oral bone regeneration have to deal with a specific environment, e.g., through saliva during wound closure. Salivary fluids contain approximately 99% water, a variety of electrolytes (sodium, potassium, calcium, Cl^−^), proteins, glucose, and nitrogenous products, such as urea and ammonia [81]. Saliva contains less Cl^−^ amounts than blood serum. According to the corrosion of Mg previously described, Mg(OH)_2_ is deposited on the Mg matrix and forms a protective layer when Mg and its alloys are in contact with salivary fluids. Additionally, the high number of electrolytes and proteins present in the saliva interact with the Mg-based materials and influence the corrosion rate. A recent in vitro study demonstrates that prior artificial exposure significantly decreases the corrosion rate and can act as a protective element directly after exposure in the oral cavity [82]. Additional preclinical research has to be performed.

Oral and maxillofacial bioresorbable implants require a homogenous corrosion associated with a corrosion rate in accordance with the bone healing in order to prevent early implant failure and negative side effects. According to Table 1, bioresorbable Mg-based alloys could be promising candidates for bone regeneration approaches within maxillo-facial and dento-alveolar surgery.

## 3. Clinical Evaluation of Mg-Based Alloys

Mg and its alloys have been widely investigated in the last century in many fields of applications such as orthopaedic, maxillo-facial, or vascular surgery. This review only focuses on the recent clinical/functional outcomes of Mg in osteosynthetic applications. Until now, no clinical trial reported the use of Mg and/or its alloys in oral bone regeneration.

### 3.1. Renewed Interest in the Use of Mg in Orthopaedic Surgery during the 20th Century

Metallic non-bioresorbable biomaterials such as Ti or cobalt-chromium are widely used in orthopaedic surgery and provide a stable fixation for fracture fixation. Their wide application is limited due to the stress conduction during bone healing, the need for a second removal procedure, and the generation of toxic metallic ions [83,84].

As summarized in Table 2, Mg and its alloys was intensively investigated through clinical orthopaedic trials including case reports, case series, retrospective observational studies, and prospective controlled clinical trials from 2013 until now. Most of these trials investigated the use of screws to stabilize unstable fractures.

#### 3.1.1. Mg and Its Alloys Studied in Orthopaedic Surgery

Concerning the type of material, most of these trials investigated the use of MgYREZr alloy. MgYREZr screws were initially investigated for hallux valgus and medial malleolar fracture [85,88]. MgYREZr pins were evaluated for osteochondritis dissecans lesions and displaced osteochondral fragments [102,110].

Despite the fast in vivo degradation, some trials investigated the use of pure Mg in the femoral head [86,89,106] and in the metatarsus [101]. ZX00, a lean Mg-Ca-Zn was also evaluated in a prospective pilot study for medial malleolar fractures [108]. Another alloy made with Mg-Ca-Zn, was studied as bioresorbable Mg alloy screws for hand fractures requiring internal fixation [87]. 

Recently, Xie et al. used calcium phosphate coated Mg-Nd-Zn-Zr screw for medial malleolar fractures [109]. This was the first clinical trial investigating a coating on Mg implant.

#### 3.1.2. Associated Complications

According to a recent systematic review and meta-analysis including eight studies (three randomized clinical trials [85,89,94], one retrospective study [109], two case-control studies [98,103], and two prospective studies [88,111]) and involving a total number of 230 patients, the estimated complication rate was 13.3% for the group treated with Mg screw [112]. The meta-analysis did not show any significant difference for complications between the use of Mg and Ti screws [112]. Additionally, one case series revealed an extensive cystic lesion after Mg implantation in an unstable scaphoid fracture [93]. Wichelhaus et al. described complications in one subject with a scaphoid fracture associated with a scaphotrapezotrapezoidal arthritis treated with a MgYREZr screw [90]. The patient complained about a painful paraesthesia in the dorsal thumb region and a subcutaneous gas accumulation [90].

**Figure 1:**
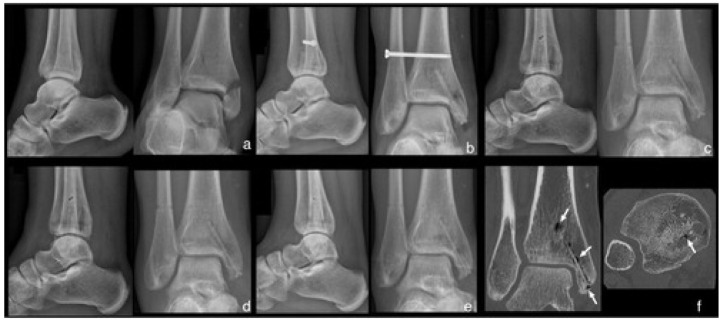
(**a**) Anteroposterior and lateral ankle radiographs of a 49-year-old female patient with medial malleolar fracture treated with Mg-based screws. (**b**) After 2 weeks: visible fracture line at the medial malleolus associated with small signs of radiolucent zones within the bone surrounding the screws. (**c**) After 6 weeks: fracture consolidation with increase of radiolucent zones within the bone surrounding the screws. (**d**) After 12 weeks, and (**e**) After 24 weeks: constant and non-evolutive radiolucent zones. (**f**) CT scan at 52 weeks, decrease of radiolucent zones, increased endosteal bone mass, and periosteal bone ingrowth at the screw head (white arrow). Reproduced from [108].

Upon resorption of Mg and its alloys, corrosion products such as H_2_ are released in the local environment. This is characterized radiographically by the appearance of radiolucent zones (Figure 1). These zones are localized around the screws and slightly increase during the first 6 months after implantation. However, radiolucent zones decreased afterwards and were not related to complications [85,87,90,93,101,105,107]. Physicians and surgeons have to be aware of this radiographical aspect of product accumulation around the implanted Mg-based material as well as the rare possibility of subcutaneous gas accumulation. This last complication is infrequent and no case of subcutaneous gas accumulation was reported in a systematic review including 230 Mg-based screws [112].

Upon resorption, Mg and its alloy release Mg^2+^ ions, which are either stored in bone or released into the circulation, thereby potentially resulting in the manifestation of hypermagnesemia. However, Holweg et al. demonstrated the normal range of Mg concentration in the blood up to 12 weeks after Mg-based screw fixation in 20 patients [108]. This Mg blood level assessment was confirmed by two other trials [85,89].

#### 3.1.3. Screw Removal

Hardware removal with non-resorbable Ti implants are frequent and estimated at around 8% for hallux valgus surgeries [113] to approximately 80% in case of ankle fractures [114,115]. Biber et al. reported the removal of one Mg screw 8 months after surgery because of radiolucency around the screw. Yu et al. also communicated a hip replacement one year after surgery due to the non-union of a femoral neck fracture stabilized with a pure Mg screws [86].

According to these promising clinical results, the use of Mg as a bioresorbable material may be an alternative to Ti for load bearing applications.

### 3.2. Application of Mg-Based Implants in the Field of Orthognathic Surgery

Implantation of Mg-based screws in the maxillo-facial region was investigated in two clinical studies conducted by Leonhardt H et al. As exposed in Table 3, compressive MgYREZr screws were used to treat condylar fractures [111,116]. The temporomandibular joint was well-restored in all patients with satisfactory improvement of the mandibular movements (laterotrusion, protrusion). In one subject, one screw was removed after a shock during an epileptic seizure [116]. In the most recent retrospective study, the screw penetrated through the condylar surface in one patient. However, no screw removal was necessary [111]. Radiolucent zones around the screws, due to the degradation products (H_2_), were also found in the mandibular condyle after implantation without screw-related complication [111,116].

Mg and its alloys are promising materials for osteo-synthetic application and showed very promising results concerning the effectiveness and the safety of their use. However, the heterogeneity between the clinical trials is significant, with only a few clinical trials such as randomized controlled trials with a high level of evidence.

## 4. Bioresorbable Synthetic Materials in Oral Bone Regeneration: Limitations and Perspective Concepts

### 4.1. Use and Limitations of Current Synthetic Bioresorbable Materials in Bone Regeneration

Among synthetic biomaterials, synthetic bioceramics and polymers were widely evaluated in the field of bone tissue engineering. Calcium phosphate, hydroxyapatite (HA), alpha (α)- and beta (β)-tricalciumphosphates (TCP) classified into synthetic bioceramics are well-documented as bone grafts because of their biocompatibility, bioresorbability, and osteoconductivity [117,118]. Pure HA (C_10_(PO_4_)_6_(OH)_2_), a principal component of bone, is available in different forms. As solid block form, HA has a much higher modulus of elasticity than bone and does not permit fibro-osseous ingrowth [119]. β-TCP is available in porous or solid form, allowing a multitude of clinical applications [119]. The combination of these two phases (TCP and HA), also called biphasic calcium phosphate, provides an excellent biocompatible and osteoconductive potential, especially the form with 40% TCP and 60% HA [120,121]. In parallel, bioceramics combined with additive manufacturing procedures have been developed and have shown osteogenic properties while being designated to match perfectly with the bone defect [122,123]. The main disadvantage of synthetic bioceramics is the poor mechanical properties characterized by a fragility of the material [124,125]. Additionally, the degradation rate of these alloplasts is difficult to predict. Concerning β-TCP, a complete absorption is unpredictable associated with some remaining ceramic residues [126,127]. Bioceramics are used commercially in various applications like coatings for biomedical implants [128].

Synthetic polymers have promising properties to be used for bone tissue engineering approaches due to their biodegradability and biomechanical properties. Poly-L-lactic acid (PLLA) and co-polymers poly(lactic-co-glycolic acid) (PLGA), the most common synthetic polymers, are degraded through hydrolysis of the ester bonds [129,130]. Due to their inherent thermoplastic properties, these polymers can easily be tailored in different shapes [131]. Despite the interesting possibility to release drugs such as antibiotics, their degradation release acidic compounds which could be compromising on bone [132,133]. Additionally, some concerns have been reported concerning the osteoconductivity of synthetic polymers and the local pH alterations during the degradation [134].

### 4.2. Advantages of Bioresorbable Mg-Based Alloys

Bioresorbable Mg-based alloys have some biomechanical primacy due to their human bone-like mechanical strength, which offers an advantage to synthetic bioceramics and polymers [135]. With a similar Young’s Modulus to that of bone, these alloys minimize the stress shielding effect in load-bearing applications. This stress is a direct consequence of the change in load to the bone after implant placement that triggers the resorption of surrounding bone tissue according to Wolff’s laws [136]. Radiographically, this stress can be highlighted by radiolucent areas.

Beside the favourable mechanical properties, Mg and its alloys aim to degrade in the physiological environment while being mechanically stable at the early stage of implantation [62]. Alloys based on Mg have not only the property to degrade in the physiological environment, these alloys manifest compatibility with living tissues without any toxic, injurious, or immunologic response. As exposed, Mg-based alloys promote bone formation after in vivo implantation in small and large animals.

One of the big disadvantages of the other synthetic polymers is the unpredictable degradation rate. Recent advancement in alloy development, as well as the progress on surface treatments and coatings, permit the reduction of corrosion rate. Mechanical surface treatment such as laser peening, actively decrease the corrosion rate of Mg and it alloys [137,138]. During the laser peening, expanding plasma induces deep compressive residual stresses which increase fatigue strength and corrosion resistance of the material [138]. Apart from that, other strategies such as surface modification using chemical treatment or coatings by single layer or multilayers have been developed to control the corrosion and degradation while keeping the biocompatibility of Mg [139].

Some Mg alloys were produced by forced molten Mg alloys into a mould cavity under high pressure. This production is called casting, especially die-castings [1,2]. However, cast alloys showed a defective microstructure associated to poor mechanical properties [2]. One attribute of Mg is its extrudability. Mg alloys have a low extrudability, i.e., extrusion is possible under lower speeds and within a narrower range of extrusion temperatures [140]. Conventional and special shapes of the extruded Mg can be designed with considerable freedom. Additionally, the extrusion process is easy to perform with a high surface quality and microstructure [141]. Recently, advances in additive manufacturing (AM) commonly known as 3D printing, offer the possibility to fabricate porous Mg scaffolds with complex shape and geometry. The benefit of AM is to fully control topological parameters and thus to fabricate interconnected porous structures. A challenge which increases when the desired size scale gets smaller [142]. AM production of Mg-based scaffold is still limited and in development as only a small number of research groups are involved in this field [142]. As Mg is highly flammable, a major associated concern and challenge is the safety in operation. During scaffold’s fabrication with the selective laser melting technique, which is the most chosen AM technique to produce Mg scaffold, devices need a high-power laser to melt the powder due to the high reflectivity of Mg [143].

Finally, Mg exhibits antimicrobial activity again *Escherichia coli*, *Pseudomonas aeruginosa*, and *Staphylococcus aureus,* which are able to form biofilms and intrinsically linked with oral infections [144,145,146].

### 4.3. Perspectives and Challenges

According to their, Mg-based alloys seem to be potential candidates for oral, maxillo-facial trauma, and orthopaedic surgery approaches. Due to the excellent osteoconductivity and biocompatibility, Mg and its alloys could be used as pure synthetic bone graft materials as well as osteosythentic indication. As observed in pre-clinical studies, the material is gradually replaced by newly formed bone [52,54,57,61,63,64]. In addition, recent efforts on AM techniques permit the fabrication of porous scaffold made with Mg-based alloys. With its advantages of rapid prototyping, digitalization, and customization, the AM technologies can effectively meet the needs of personalized medical devices in oral bone regeneration by matching the 3D printed implant with the bone defect. Customization to the scaffold includes optimization of the inner structures to ameliorate the bone in-growth. Mg-based alloys could be also used as a barrier membrane during the GBR/GTR procedures.

In the field of orthognathic surgery, bioresorbable screws were investigated in cases of mandibular condyle fracture with promising clinical and functional outcomes [111,116]. Recently, preclinical study with miniature pigs model aimed to develop plates and screw for mandibular, zygomatic, and orbital fracture treatment [147]. However, due to the thickness and the surface of the devices necessary to permit a mechanical stability and the pronounced H_2_ production for implants with large surface, released H_2_ may impact their applicability.

## 5. Conclusions

Mg and its alloys demonstrate excellent bioresorbable, osteoconductive, and antibacterial properties for an ideal future application in the field of oral bone regeneration. Clinical evaluation in the field of orthopaedic and orthognathic surgeries demonstrates the effectiveness and safety of Mg-based bioresorbable implants. For intra-oral use, additional variables need to be understood, such as the effect of saliva on Mg. Finally, long-term pre-clinical and clinical studies are warranted to understand (i) the time of complete degradation of the bioresorbable devices, (ii) the associated clinical degradation rate, and (iii) the related tissue replacement after the complete degradation.

## Figures and Tables

**Table 1:** Representative pre-clinical studies of Mg-based biomaterials.

Type of Material	Designed Material	Animals and Implantation Site	Time Period	Calculated In Vivo Degradation Rate
Pure Mg (99.99 wt.%) [49]	plate and screw	Rabbit ulnae	8 and 16 weeks	0.40 ± 0.04 mm/y (8 weeks)
Pure Mg (99.99 wt.%) [50]	screw	Rabbit femoral bones	4, 8, 16, 24 weeks	1.38 ± 0.03 mm/y(4 weeks) 0.57 ± 0.03 mm/y (24 weeks)
Pure Mg (99.99 wt.%) [51]	pin	Rat femoral bones	1, 4 and 12 weeks	0.2–0.4 mm/y
Pure Mg (99.99 wt.%) [52]	pin	Rat femoral bones	7 days	0.15 ± 0.03 mm/y
^26^Mg enriched (>99 wt.%) [53]	pin	Rat femoral bones	4, 24, 52 weeks	16 ± 5 µm/y
Pure Mg high pressure (99.98 Mg in wt.%) [54]	disk	Rat femoral bones	24 weeks	0.41 ± 0.02 mm/y
WE43 (>92.0 Mg in wt.%) [55]	plates and screw	Dogs-LeFort I osteotomy	4, 12, and 24 weeks	NA ^1^
WE43 (>92.0 Mg in wt.%) [56]	screw	Rabbit tibiae	4, 8, 12 and 16 weeks	NA ^1^
WE43 (>92.0 Mg in wt.%) [57]	pin	Rat tibiae	52 weeks	NA ^1^
WE43/WE43T5 (>92.0 Mg in wt.%) [58]	screw	Sheep mandibule	6 and 24 weeks	NA ^1^
MgYREZr (>92.0 Mg in wt.%) [59]	screw	Rabbit femoral bones	1, 12, and 52 weeks	NA ^1^
JDBM (>95.0 Mg in wt.%) [60]	screw	Rabbit mandible bones	1, 4, and 7 months	0.161 ± 0.025 mm/y (1 month), 00.218 ± 0.030 mm/y (7 months)
NZK (>96.0 Mg in wt.%) [61]	rod	Rabbit femoral bones	28 and 56 days	0.66 mm/y (28 days) and 0.48 mm/y (56 days)
Mg-Zn (94.0 Mg in wt.%) [62]	rod	Rabbit femoral bones	14 weeks	2.32 mm/y
ZX00 (>99.0 Mg in wt.%) [63]	pin	Rat femoral bones and sheep tibiae	6, 12 and 24weeks	0.08 mm/y (rat) and 0.045 mm/y (sheep)
ZX00 (>99.0 Mg in wt.%) [64]	screw	Sheep tibiae	3, 6 and 12 weeks	NA ^1^

^1^ NA: non-applicable for the degradation rate in mm/year. JDBM, Jiaoda BioMg, denoted as JDBM; NZK, Mg-Nd-Zn-Zr alloy.

**Table 2:** Summary of the clinical studies using Mg-based implant in the field of orthopaedic surgery and traumatology.

Authors	Type of Study	Intervention	Type of Alloy	*n*	Outcomes	Complications
Windhagen et al. [85]	Randomized controlled clinical trial	Hallux valgus	MgYREZr	26 (13 Mg and 13 Ti)	Fracture union without healing disorders except for 2 patients	Wound healing delayed in 2 patients with Mg screws
Yu X et al. [86]	Retrospective observational study	Displaced femoral neck fracture	Pure Magnesium	19	17 patients with fracture union	One patient with non-union fracture
Lee et al. [87]	Case series	Radial styloid fracture	Mg-5wt%Ca-1wt%Zn	53	Fracture union, fracture healing, retrieved function	None
Plaass C et al. [88]	Case series	Hallux valgus	MgYREZr	45	Fracture union, fracture healing, retrieved function	One complication (dorsal subluxation)
Zhao et al. [89]	Randomized controlled clinical trial	Hip-preserving surgery	Pure Magnesium	48	Fracture union, fracture healing, retrieved function	None
Wichelhaus A et al. [90]	Case report	Scaphotrapeziotrapezoidal fracture	MgYREZr	1	/	Screw breakage, pain, paraesthesia
Biber R et al. [91]	Case report	Humeral capitelum fracture	MgYREZr	1	Fracture union, fracture healing, retrieved function	None
Biber R et al. [92]	Case report	Distal fibular fracture	MgYREZr	1	Fracture union, fracture healing, retrieved function	None
Meyer R and Panzica M [93]	Case series	Scaphoid fracture	MgYREZr	5	/	Extensive cyst formation and delayed consolidation
Plaass C et al. [94]	Randomized controlled clinical trial	Hallux valgus	MgYREZr	26 (13 Mg and 13 Ti)	Fracture union, fracture healing, retrieved function	None
Kose O et al. [95]	Retrospective observational study	Medial malleolar fracture	MgYREZr	11	Fracture union, fracture healing, retrieved function	None
Acar B. et al. [96]	Retrospective observational study	Hallux valgus	MgYREZr	31 (16 Mg and 15 Ti)	Fracture union, fracture healing, retrieved function	One delayed wound healing -Mg screw
Choo JT et al. [97]	Randomized controlled clinical trial	Hallux valgus	MgYREZr	93 (24 Mg and 69 Ti)	Fracture union, fracture healing, retrieved function	3 superficial cellulitis and one neuropathic operative site pain.
Klauser H. [98]	Retrospective observational study	Hallux valgus	MgYREZr	200 (100 Mg and 100 Ti)	Fracture union, fracture healing, retrieved function	None
Gigante A. et al. [99]	Case series	Anterior cruciate ligament avulsion fracture	MgYREZr	3	Fracture union, fracture healing	None
Acar B. et al. [100]	Case report	Lateral malleolar fracture	MgYREZr	1	Fracture union, fracture healing, retrieved function	None
Kim, Y.-K et al. [101]	Retrospective observational study	Meta-tarsal or midfoot fractures	Pure Magnesium	22	Fracture union, fracture healing	2 wound dehiscence
Aktan C et al. [102]	Case report	Distal humerus intra-articular fracture	MgYREZr	1	Fracture union, fracture healing, retrieved function	None
Atkinson HD et al. [103]	Randomized controlled clinical trial	Hallux valgus	MgYREZr	36 (11 Mg and 25 Ti)	Fracture union, fracture healing, retrieved function	None
Acar B et al. [104]	Randomized controlled clinical trial	Biplane chevron medial malleolar osteotomy	MgYREZr	22	Fracture union, fracture healing, retrieved function	None
Turan A. [105]	Case report	Radial styloid fracture	MgYREZr	2	Fracture union, fracture healing, retrieved function	None
Chen L et al. [106]	Case report	Traumatic femoral head necrosis	Pure magnesium	1	Improvement of patient’s hip function	None
May H et al. [107]	Retrospective observational study	Medial malleolar fracture	MgYREZr	48 (25 Mg and 23 Ti)	Fracture union, fracture healing, retrieved function	None in the Mg group
Holweg et al. [108]	Case series	Medial malleolar fracture	Mg-Zn0.45-Ca0.45, in wt. (ZX00)	20	Fracture union, fracture healing, retrieved function	None
Xie K et al. [109]	Case series	Medial malleolar fracture	Mg-Nd3.0-Zn0.2- Zr0.5 in wt. and coating	9	Fracture union, fracture healing, retrieved function	None
Jungesblut et al. [110]	Retrospective observational study	Osteochondritis dissecans lesion	MgYREZr	19	Fracture union, fracture healing	One post-operative implant failure

**Table 3:** Summary of the clinical studies investigating Mg-based implant in the field of maxillo-facial surgery.

Authors	Type of Study	Intervention	Type of Device	*n*	Outcomes	Complications
Leonhardt H et al. [116]	Case series	Mandibular fracture	MgYREZr	5	Fracture healing, with restored function of the temporomandibular joint	One fracture of a screw
Leonhardt H et al. [111]	Retrospective observational study	Mandibular fracture	MgYREZr	6	Fracture healing with restored function of the temporomandibular joint	Penetration of one screw tip through the condylar surface without screw removal necessary
